# Tonsillectomy Combined With Steroid Pulse Therapy Prevents the Progression of Chronic Kidney Disease in Patients With Immunoglobulin A (IgA) Nephropathy in a Single Japanese Institution

**DOI:** 10.7759/cureus.15736

**Published:** 2021-06-18

**Authors:** Sae Aratani, Takeshi Matsunobu, Akira Shimizu, Kimihiro Okubo, Tetsuya Kashiwagi, Yukinao Sakai

**Affiliations:** 1 Nephrology, Graduate School of Medicine, Nippon Medical School, Tokyo, JPN; 2 Division of Cancer Cell Biology, Institute of Medical Science, University of Tokyo, Tokyo, JPN; 3 Otolaryngology-Head and Neck Surgery, Graduate School of Medicine, Nippon Medical School, Tokyo, JPN; 4 Analytic Human Pathology, Graduate School of Medicine, Nippon Medical School, Tokyo, JPN; 5 Otolarynogology-Head and Neck Surgery, Graduate School of Medicine, Nippon Medical School, Tokyo, JPN

**Keywords:** iga nephropathy, tonsillectomy, chronic kidney disease, urinary protein

## Abstract

Background

Despite the abundant experience of tonsillectomy with steroid pulse therapy (TSP) for patients with immunoglobulin A (IgA) nephropathy, the therapeutic efficacy of TSP on renal prognosis remains controversial. The purpose of this study was to evaluate the efficacy of whether TSP effectively prevents chronic kidney disease (CKD) progression.

Methods

This was a single-center, retrospective observational study. A total of 149 patients were enrolled in the current study who were confirmed with IgA nephropathy by renal biopsy between February 2011 and August 2019. The impact of TSP on CKD progression was compared with conservative treatment during a follow-up period of 3 years.

Results

In total, 110 patients received TSP and 39 patients received conservative treatment. There were no differences between the two groups in the initial CKD stages: 65.1% of patients had CKD G1-2, 32.2% had CKD G3, and 2.7% had CKD G4-5. The initial urine protein was 0.7 g/gCr, which was not different between the two groups. Kaplan-Meier analysis showed that patients with TSP had a significantly better renal prognosis than those in the conservative treatment group after one and a half years (p = 0.007). Multivariable analysis revealed that TSP had a significant impact on the prevention of CKD progression, with an adjusted odds ratio of 0.07 (95% confidence interval, 0.01-0.87; p=0.039). However, we could not confirm the predictive value of the Oxford Classification on TSP efficacy. Additionally, the initial urinary protein level was a risk factor for CKD progression.

Conclusions

TSP was associated with a lower risk of CKD progression. In this regard, our study supports that TSP may be a reasonable treatment option for patients with IgA nephropathy. In the featured study, it needs to be elucidated which histopathological classifications benefit from TSP treatment.

## Introduction

Immunoglobulin A (IgA) nephropathy was first described in 1968 by Berger [1] and is now recognized as the most common primary glomerulonephritis worldwide [2].

The pathogenesis of IgA nephropathy is uncertain; however, a plausible hypothesis is a multi-hit mechanism in which mesangial deposition of IgA1 may be the hallmark and a final common endpoint for pathogenesis. The synthesis of galactose-deficient IgA1 (Gd-IgA1) is disproportionally increased in patients with IgA nephropathy [3-4]. This aberrant Gd-IgA1 can form complexes with glycan-specific autoantibodies, resulting in pathogenic IgA1-containing circulating Gd-IgA1 immune complexes (Gd-IgA1-ICs) [5]. The Gd-IgA1-ICs deposit in the mesangial area of the glomeruli lead to the development of mesangial proliferation or matrix expansion and subsequently cause tubulointerstitial injury. Emerging evidence indicates that B cells arising in response to mucosal infection, especially tonsillitis, might generate nephritogenic IgA1 [6]. Clinical studies have also suggested that mucosal infection, especially tonsillitis, might be associated with the development of IgA nephropathy [7].

Renal prognosis is the most serious concern in patients with IgA nephropathy. The clinical symptoms of IgA nephropathy are highly heterogeneous. Symptoms range from proteinuria, microscopic hematuria, and hypertension alone or in combination with chronic kidney disease (CKD) or end-stage kidney disease (ESKD). Importantly, the natural course of renal prognosis is now recognized to be far from benign in many patients. Progression to ESKD is reported to be approximately 30%-40% over 20 years after diagnosis [8]. Therefore, various treatment options have been investigated to achieve better renal outcomes, including corticosteroids and immunosuppressive therapies such as azathioprine, calcineurin inhibitors, cyclophosphamide, and rituximab [9-12].

Tonsillectomy combined with steroid pulse therapy (TSP) is one of the available therapeutic options that has been conducted since 1988 in Japan [13]. As mentioned above, tonsillitis and the subsequent generation of nephritogenic IgA1 may contribute to the pathogenesis of IgA nephropathy. Therefore, TSP seems to be a rational strategy for pathogen-targeted treatment. However, the efficacy of TSP remains controversial [14]. Herein, we hypothesized that elucidating the therapeutic efficacy of TSP in CKD progression will further aid physicians in providing IgA nephropathy patients with better renal outcomes.

## Materials and methods

Aims

This study aimed to determine whether and how the therapeutic efficacy of TSP in CKD progression appears in patients with IgA nephropathy. We conducted this study to provide physicians with additional information regarding the treatment options for IgA nephropathy.

Setting and study patients

This single-center retrospective cohort study was conducted by reviewing the medical records of 877 beds in a university hospital (Nippon Medical University Hospital) in Japan. The study protocol complied with the Declaration of Helsinki. We included consecutive patients who underwent renal biopsy between February 2011 and August 2019. Inclusion criteria were: all the patients who were confirmed with IgA nephropathy by renal biopsy during the period were enrolled. Exclusion criteria were: patients who had received treatment previously, who received steroid therapy only, or who underwent tonsillectomy alone were ruled out.

Treatment protocol 

All patients in this study underwent tonsillectomy with steroid therapy (TSP) or conservative treatment. Physicians were responsible for the treatment. TSP fundamentally complied with the treatment protocol described by Pozzi et al. [10]. The patients in the TSP group underwent tonsillectomy and subsequently received methylprednisolone (0.5 g) intravenously for three consecutive days at one to three weeks after the surgery, one to three times. Subsequently, the patients took oral prednisolone at an initial dose of 0.5 mg/kg every other day for six months, with a gradual decrease in dosage from six months to one year. If necessary, the patients in both groups were administered anti-hypertensive drugs, which included RAS inhibitors such as angiotensin receptor antagonist (ARB) or angiotensin-converting enzyme inhibitor (ACE-I). All patients were educated about appropriate control of blood pressure, glycemic state, body weight, and diet according to CKD stages.

Primary endpoint

**Figure 1:**
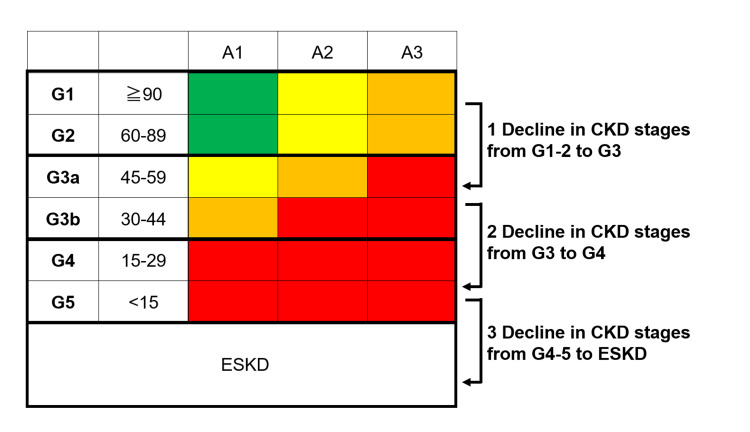
Primary endpoint Primary endpoints were set as a progression of CKD stages. We set three decline points in CKD stages: 1, the decline in CKD from G1-2 to G3; 2, from G3 to G4; and 3, from G4-5 to ESKD (initiating renal replacement therapy). CKD, chronic kidney disease; ESKD: end-stage kidney disease

CKD stages were defined according to the Kidney Disease: Improving Global Outcomes (KDIGO) guidelines [15]. We defined the primary endpoint as CKD progression. We set three points of decline in CKD stages: 1) the decline in CKD from G1-2 to G3, 2) from G3 to G4, and 3) from G4-5 to ESKD (initiating renal replacement therapy). The follow-up period was set at three years (1095 days). See Figure 1.

Statistical analysis

Categorical data were represented as percentages (%) and continuous data as medians (25th-75th percentiles). The incidence of the primary endpoint was evaluated using Kaplan-Meier analysis. We then applied landmark analysis at the 1.5-year (550 days) mark, the time when differences started to appear between the two groups. Finally, differences were evaluated using the log-rank test. The impact of patient parameters on the incidence of CKD stage progression was estimated using univariable and multivariable Cox regression analysis and represented as odds ratios (OR) with 95% confidence intervals (95% CI). In the multivariable model, we selected covariates to be included in the analysis according to the clinical utility. In addition, we applied the Akaike Information Criteria (AIC) to use appropriate covariates in the multivariable model. The AIC is a mathematical method to evaluate how well a model fits the data it is meant to describe. In statistics, AIC is used to compare different possible models and determine which one is the best fit for the data. For the comparison of patient parameters, we divided our patients into two groups: the TSP group (n = 110) and the conservative treatment group (n = 39). Pearson's chi-squared test or Fisher's exact test and the Wilcoxon rank-sum test were used to compare categorical data and continuous data between the groups, respectively. All statistical analyses were conducted using the R software package (version 3.5, R Development Core Team, https://www.r-project.org/).

## Results

Patient characteristics

**Figure 2:**
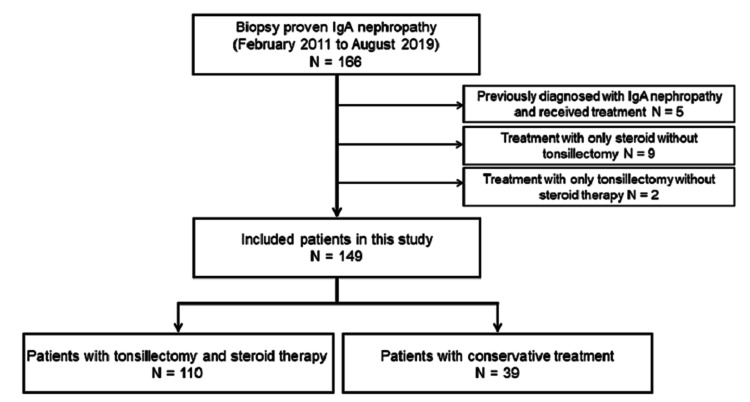
Algorithm of the study design CKD, chronic kidney disease; ESKD, end-stage kidney disease

A total of 166 patients were diagnosed with IgA nephropathy (Figure 2).

Eleven patients were excluded. Five of 11 patients had been previously diagnosed with IgA nephropathy and had already received treatment. Nine patients received only steroid therapy without tonsillectomy, and two patients underwent only tonsillectomy. One patient was discharged without follow-up. A total of 149 patients were included in this study.

Table 1 shows the characteristics of the study population with the diagnosis of IgA nephropathy. The median age was 37 years and 46 years in the TSP and conservative groups. The two groups did not differ in initial blood analysis, CKD stage, urine protein, or use of ARB or ACE-I. In total, 65.1% of patients had CKD G1-2, 32.2% had CKD G3, and 2.7% had CKD G4-5, and the initial urine protein level was 0.7 g/gCr.

**Table 1:** Patient characteristics Categorical variables are shown as numbers (percentages) and continuous variables as medians (25–75 percentiles). M1 represents a mesangial hypercellularity score of more than 0.5, E1 represents the presence of endocapillary hypercellularity, S1 represents the presence of segmental glomerulosclerosis, T1/2 represents tubular atrophy/interstitial fibrosis involving a cortical area of more than 25%, and C1 and C2 represent a crescent in at least one glomerulus or crescents in at least 25% of glomeruli, respectively. BMI, body mass index; BUN, blood urea nitrogen; Cr, creatinine; eGFR, estimated glomerular filtration rate; Hb, hemoglobin; Alb, albumin; T-cho, total-cholesterol; HbA1c, hemoglobin A1c; IgA, immunoglobulin A; C3, complement 3; C4, complement 4; RBC, red blood cell; ARB, angiotensin receptor antagonist; ACE-I, angiotensin-converting enzyme inhibitor; TSP, tonsillectomy combined with steroid pulse therapy.

Parameters	Total (n = 149)	TSP (n = 110)	Conservative treatment (n = 39)	p-value
Age, years old	39 (28-50)	37 (27-47)	46 (32-60)	0.004
Male, n (%)	69 (46.3)	52 (47.3)	17 (43.6)	0.692
Body Weight, kg	60.0 (54.0-69.0)	61.0 (54.3-69.8)	57.0 (52.5-68.0)	0.088
BMI	22.9 (20.2-24.8)	23.3 (20.4-24.7)	21.6 (19.8-25.1)	0.422
Systolic Blood Pressure, mmHg	110 (104-122)	113 (104-124)	110 (102-122)	0.334
Hypertension, n (%)	42 (28.2)	29 (26.4)	13 (33.3)	0.406
Diabetes, n (%)	8 (5.4)	4 (3.6)	4 (10.3)	0.207
Blood analysis				
BUN, mg/dL	15.3 (12.5-19.0)	15.5 (13.1-19.0)	14.4 (10.8-19.5)	0.393
Cr, mg/dL	0.9 (0.7-1.1)	0.9 (0.7-1.1)	0.9 (0.7-1.1)	0.639
eGFR, ml/min/1.73m2	74.0 (52.0-90.0)	74.5 (57.0-94.0)	64.0 (46.5-78.5)	0.057
Hb, mg/dL	13.4 (12.3-14.5)	13.6 (12.3-14.6)	13.1 (12.3-13.7)	0.124
Alb. mg/dL	4.1 (3.7-4.3)	4.1 (3.7-4.4)	4.0 (3.7-4.1)	0.082
T-Cho, mg/dL	196 (169-227)	201 (170-227)	179 (159-214)	0.077
HbA1c	5.4 (5.2-5.6)	5.4 (5.2-5.6)	5.5 (5.3-5.6)	0.108
IgA	305 (246-371)	305 (246-361)	311 (241-437)	0.599
C3	99 (88-113)	99 (87-111)	103 (91-115)	0.276
C4	25 (21-30)	25 (21-30)	25 (22-28)	1.000
Urinalysis				
Proteinuria, g/gCr	0.7 (0.2-1.6)	0.7 (0.3-1.6)	0.6 (0.2-1.5)	0.272
RBC <4/HPF, n (%)	6 (4.0)	1 (0.9)	5 (12.8)	0.005
RBC 5-19/HPF, n (%)	40 (26.9)	27 (24.5)	13 (33.3)	0.287
RBC 20-49/HPF, n (%)	46 (30.9)	34 (30.9)	12 (30.8)	0.987
RBC >50/HPF, n (%)	57 (38.2)	48 (43.6)	9 (23.1)	0.023
CKD Stages				
Stage G 1-2, n (%)	97 (65.1)	76 (69.1)	21 (53.8)	0.086
Stage G 3, n (%)	48 (32.2)	31 (28.2)	17 (43.6)	0.077
Stage G 4-5, n (%)	4 (2.7)	3 (2.7)	1 (2.6)	1.000
Oxford Classification				
M1, n (%)	37 (24.8)	31 (28.2)	6 (15.4)	0.181
E1, n (%)	60 (40.3)	53 (48.2)	7 (17.9)	0.002
S1, n (%)	106 (71.1)	82 (74.5)	24 (61.5)	0.122
T1/T2, n (%)	12 (8.1)	8 (7.3)	4 (10.3)	0.694
C1/2, n (%)	11 (7.4)	9 (8.2)	2 (5.1)	0.094
Medication				
ARB/ACE-I, n (%)	69 (46.3)	49 (44.5)	20 (51.3)	0.469
Statin, n (%)	31 (20.8)	24 (21.8)	7 (17.9)	0.609

CKD progression

**Figure 3:**
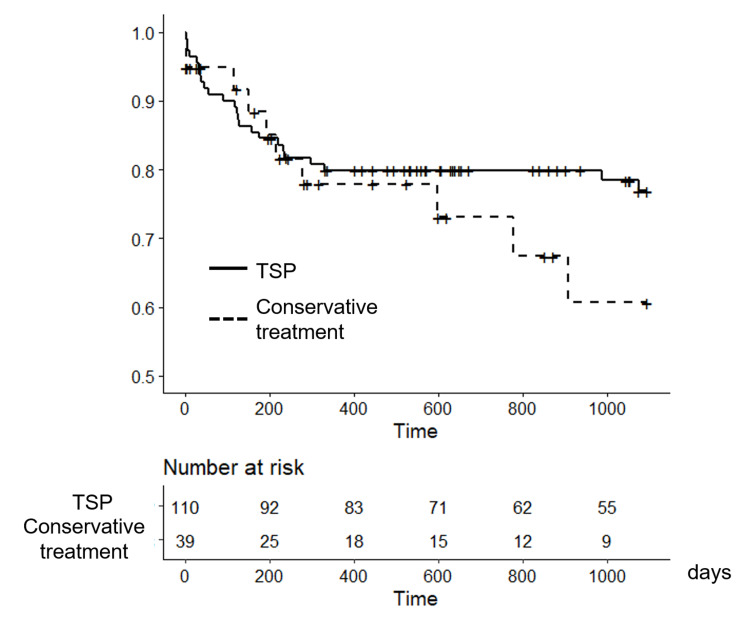
Comparison of CKD progression between the TSP and conservative treatment groups Kaplan–Meier curves signiﬁcantly differ (p = 0.007; log-rank test). CKD, chronic kidney disease; TSP, tonsillectomy with steroid pulse therapy

Figure 3 shows the incidence of the primary outcome. Notably, during the first year and a half (550 days), there were no differences between the two groups. After this period, patients with TSP showed a significantly better renal prognosis than those in the conservative treatment group (p = 0.007).

Risk factor of renal progression

Regarding the risk of the primary outcome, Table 2 shows that TSP had a better impact on renal prognosis than conservative treatment. The initial age, estimated glomerular filtration rate (eGFR), early CKD (CKD 1-2), urine red blood cell count, urine protein level, and Oxford Classification M, S, and T score did not significantly affect renal prognosis in the univariate analysis.

**Table 2:** Impact of each variable on CKD progression in univariable analysis CI, confidence interval; eGFR, estimated glomerular filtration rate; HPF, high power field; OR, odds ratio; and RBC, red blood cell

	Univariable analysis	
Parameters	OR (95% CI)	p-value
Treatment	0.12 (0.02-0.75)	0.023
Age	1.05 (0.95-1.11)	0.125
eGFR	0.97 (0.93-1.00)	0.111
CKD 1-2	0.37 (0.06-2.24)	0.282
Urine RBC >50/HPF	0.40 (0.40-3.57)	0.411
Proteinuria	1.37 (0.86-2.16)	0.182
Oxford Classification M1	0.69 (0.08-6.22)	0.744
Oxford Classification S1	0.37 (0.06-2.22)	0.276
Oxford Classification T1/2	4.71 (0.53-42.2)	0.166

In the multivariable Model 1, we included TSP and urine protein according to the results of the AIC analysis (likelihood ratio test = 8.78, p=0.01). This demonstrated that both TSP and initial proteinuria had a significant impact on renal prognosis.

Nephropathy Classification Working Group M, S, and T scores have a predictive value [16]. In contrast, the E score has a predictive value only in patients without immunosuppression treatment. In our study, the C score was evaluated after January 2018. Therefore, only 25 patients received the C score. We included M, S, and T as predictive evaluations in multivariable Model 2. We showed that both TSP and initial proteinuria had a significant impact on renal prognosis. However, we could not identify the prognostic value of histological classification nor early CKD (CKD 1-2). See Table 3.

**Table 3:** Impact of each variable on CKD progression in multivariable analysis CI, confidence interval; CKD, chronic kidney disease; OR, odds ratio

	Multivariable analysis (Model 1)		Multivariable analysis (Model 2)	
Parameters	adjusted OR (95% CI)	p-value	adjusted OR (95% CI)	p-value
Treatment	0.05 (0.01-0.52)	0.011	0.07 (0.01-0.87)	0.039
Proteinuria	1.80 (1.07-3.03)	0.027	2.08 (1.11-3.90)	0.023
CKD 1-2	-	-	2.05 (0.16-25.49)	0.58
Oxford Classification M1	-	-	0.73 (0.06-9.71)	0.814
Oxford Classification S1	-	-	0.23 (0.02-2.47)	0.224
Oxford Classification T1/T2	-	-	6.59 (0.37-118.58)	0.201

## Discussion

In this single-center, retrospective observational study, we evaluated the therapeutic efficacy of TSP on renal prognosis compared to conservative treatment by enrolling 149 patients diagnosed with IgA nephropathy. We observed two clinically valuable observations. First, TSP was significantly more effective in preventing CKD progression than conservative treatment during the later treatment time-course. Second, initial proteinuria was an independent risk factor for CKD progression in patients with IgA nephropathy.

The tonsils are mucosal lymphatic organs and are recognized to play an important role in the pathogenesis of IgA nephropathy. The palatine tonsils are considered a major site for the production of Gd-IgA1 [14]. Moreover, plasma cells producing circulating nephritogenic polymeric IgA1 and glomerular-deposited immune complexes are suggested to form in tonsils. The serum level of Gd-IgA1 was elevated in patients with IgA nephropathy [17]. Therefore, tonsillectomy may be warranted to eradicate the sources of nephritogenic IgA1. However, conflicting results have been reported regarding the therapeutic efficacy of TSP for IgA nephropathy [18-22].

A recent large cohort study, including 1065 patients with IgA nephropathy demonstrated that tonsillectomy was associated with a lower risk of the primary endpoint and progression of kidney disease (hazard ratio, 0.34; 95% CI, 0.13-0.77; P = 0.009) [23]. According to the study, the benefit of tonsillectomy was confirmed in the complete cohort and was independent of baseline estimated glomerular filtration rate (eGFR), proteinuria, degree of hematuria, and prior use of RAS-I. In addition, they concluded that tonsillectomy was effective regardless of whether patients were treated with oral or intravenous corticosteroids during the follow-up period. A previous meta-analysis indicated that tonsillectomy might be helpful in inducing clinical remission and preventing the development of ESKD [20-21]. Another multicenter randomized controlled trial showed that TSP contributed to the disappearance of proteinuria although it did not result in clinical remission compared to TSP alone [24]. Consistent with previous results, our study supported that TSP was significantly effective in preventing CKD progression compared to conservative treatment. Of note, the beneficial effect of TSP became obvious during the later time course in our study. Therefore, it might be necessary for physicians to wait for a certain period to expect benefits from TSP for renal prognosis.

In contrast to the positive result of TSP, whether TSP is an effective treatment for IgA, nephropathy remains controversial. The European validation study of the Oxford classification of IgA nephropathy (VALIGA), enrolling 1147 European IgA nephropathy patients, reported that no significant relationship was found between tonsillectomy and renal improvement [22]. A recent network meta-analysis concluded that TSP did not have a beneficial effect on ESKD or doubling of serum creatinine levels compared to RAS inhibitor alone [25]. They indicated that RAS inhibitor with steroids was the best treatment for disease remission and long-term renal protection in patients with IgA nephropathy. Thus, the therapeutic efficacy of TSP in improving renal prognosis remains to be elucidated.

It is clinically variable that TSP may be safely applied to IgA nephropathy patients with deteriorated renal function. Although the use of corticosteroids is suggested for patients with IgA nephropathy and eGFR >50 mL/min/1.73m2 and tonsillectomy is not recommended [26], a previous report showed that TSP was safe and effective in preserving long-term renal function in patients with impaired kidney function [18]. In their study, pre-treatment eGFR was 47.1 ± 7.4 mL/min/1.73m2 in both the TSP and conservative treatment groups, and TSP contributed to a higher remission rate of hematuria compared to oral steroid therapy and a higher remission rate of proteinuria compared to conservative therapy. Moreover, TSP was associated with a better renal survival rate to a 25% decline in eGFR from baseline and prevented the progression to ESKD at 10 years in all patients. Sato et al. also conducted TSP in IgA nephropathy patients with a creatinine level of 1.5-2.0 mg/dL, which showed that TSP was superior to supportive therapy in improving the eight-year renal survival rate [27]. In our study, approximately 30% and 45% of patients in the TSP and conservative groups were more advanced than CKD stage 3, respectively. Accordingly, TSP may be a safe and beneficial treatment option for patients with IgA nephropathy and impaired renal function.

Recently, reduction of proteinuria has been regarded as a reasonable surrogate endpoint for the effect of treatment on progression to ESKD in IgA nephropathy [28]. Thompson et al. analyzed data including 13 controlled trials and showed an association between treatment effect on proteinuria reduction and treatment effect on a composite of doubling of serum creatinine, ESKD, or death. Proteinuria is the most well-studied risk indicator for progression to ESKD. A recent cross-sectional study revealed that daily proteinuria was correlated with all histological parameters, including both acute and chronic glomerular lesions, and the mesangial score [29]. Consistent with prior results, we also found that initial proteinuria was significantly related to CKD progression. Our study supports the rationale for using proteinuria as a surrogate endpoint in patients with IgA nephrology.

This study had some limitations. First, this was a single-center retrospective study with a small sample size of 149 patients. Therefore, only a few patients reached the primary endpoint in the final analysis. Second, we could not find the prognostic value of the histopathological classification. Although, the IgA Nephropathy Classification Working Group recommended describing the MEST-C score for all the patients with IgA nephropathy [16], only 25 patients who were diagnosed after January 2018 received the C score in the current study. Further study is warranted to clarify whether and which histopathological classifications are related to the benefit of TSP treatment. Third, we enrolled patients with IgA nephropathy regardless of CKD stage. This might make it more challenging to identify the CKD stages that could most appropriately and beneficially improve renal outcomes. In the current study, the early CKD stage (CKD 1-2) itself did not show the factor to prevent CKD progression. Finally, the use of ARB or ACE-I was limited to only 44.5% and 51.9% of patients in the TSP and conservative groups, respectively. Previously, intensive supportive care was not inferior to the addition of immunosuppressive therapy for a decline in eGFR among patients with high-risk IgA nephropathy [30]. In addition, the KDIGO guidelines suggested the use of RAS inhibitors with up-titration of dosage as far as tolerated [26]. In our study, the use of RAS inhibitors should have been encouraged more aggressively, and the administration of RAS inhibitors was dependent on each physician in our study. Our data should be interpreted in light of these limitations.

## Conclusions

TSP may be a clinically effective treatment option for patients with IgA nephropathy to prevent CKD progression. The current study supports the previous evidence that TSP might prevent renal disease progression. In addition, we showed the safety of TSP for IgA nephropathy patients with impaired kidney function. Notably, primary proteinuria was the most reliable marker. In a future study, pathological evaluation needs to be expanded to elucidate the histopathological population most susceptible to TSP benefit.
